# Giant Magnetoresistance and Magneto-Thermopower in 3D Interconnected Ni_*x*_Fe_1−*x*_/Cu Multilayered Nanowire Networks

**DOI:** 10.3390/nano11051133

**Published:** 2021-04-27

**Authors:** Nicolas Marchal, Tristan da Câmara Santa Clara Gomes, Flavio Abreu Araujo, Luc Piraux

**Affiliations:** Institute of Condensed Matter and Nanosciences, Université Catholique de Louvain, Place Croix du Sud 1, 1348 Louvain-la-Neuve, Belgium; nicolas.marchal@uclouvain.be (N.M.); tristan.dacamara@uclouvain.be (T.d.C.S.C.G.); flavio.abreuaraujo@uclouvain.be (F.A.A.)

**Keywords:** 3D nanowire networks, giant magnetoresistance, spin caloritronics, flexible thermoelectrics, nickel-iron alloys

## Abstract

The versatility of the template-assisted electrodeposition technique to fabricate complex three-dimensional networks made of interconnected nanowires allows one to easily stack ferromagnetic and non-magnetic metallic layers along the nanowire axis. This leads to the fabrication of unique multilayered nanowire network films showing giant magnetoresistance effect in the current-perpendicular-to-plane configuration that can be reliably measured along the macroscopic in-plane direction of the films. Moreover, the system also enables reliable measurements of the analogous magneto-thermoelectric properties of the multilayered nanowire networks. Here, three-dimensional interconnected Ni${}_{x}$Fe${}_{1-x}$/Cu multilayered nanowire networks (with 0.60≤x≤0.97) are fabricated and characterized, leading to large magnetoresistance and magneto-thermopower ratios up to 17% and −25% in Ni${}_{80}$Fe${}_{20}$/Cu, respectively. A strong contrast is observed between the amplitudes of magnetoresistance and magneto-thermoelectric effects depending on the Ni content of the NiFe alloys. In particular, for the highest Ni concentrations, a strong increase in the magneto-thermoelectric effect is observed, more than a factor of 7 larger than the magnetoresistive effect for Ni${}_{97}$Fe${}_{3}$/Cu multilayers. This sharp increase is mainly due to an increase in the spin-dependent Seebeck coefficient from −7 µV/K for the Ni${}_{60}$Fe${}_{40}$/Cu and Ni${}_{70}$Fe${}_{30}$/Cu nanowire arrays to −21 µV/K for the Ni${}_{97}$Fe${}_{3}$/Cu nanowire array. The enhancement of the magneto-thermoelectric effect for multilayered nanowire networks based on dilute Ni alloys is promising for obtaining a flexible magnetic switch for thermoelectric generation for potential applications in heat management or logic devices using thermal energy.

## 1. Introduction

Three-dimensional (3D) networks made of interconnected high-aspect ratio nanowires (NWs) are unique macroscopic nano-architectures that have raised increasing interest over the last decade [1,2,3,4]. Their robust and self-standing structure with a high degree of NW interconnectivity and high surface over volume ratio makes them attractive nano-device components for a large range of applications. For instance, they have potentials in energy harvesting and storage systems [5,6,7,8], biosensors and bio-analytical devices [9,10,11], magnetic and spintronic devices [12,13,14], and thermoelectric and spin caloritronic devices [15,16,17,18]. Direct electrodeposition into polymer template films containing networks of crossed nano-channels has proved to be a suitable technique to grow a wide variety of 3D NW networks with controllable morphologies, geometrical parameters, and sizes [1,2,12]. In addition, the polymer-NWs composite is flexible and lightweight, which are important advantages of this structure [15,16]. The composite film can be twisted or can have a different shape without damaging the high electrical and thermal interconnectivity in the large amount crossing points of the macroscopic structure [14,18].

The successful fabrication of 3D networks made of interconnected multilayered NWs, in which successive layers of ferromagnetic metal (FM) and non-magnetic metal (NM) are stacked along the NW axis, has revealed the possibility to obtain giant magnetoresistance (GMR) effects measured in the current-perpendicular-to-plane (CPP) configuration that are easily measurable along the macroscopic in-plane dimensions of the network films [15,16,19,20]. Furthermore, the configuration has also been proven to be suitable for easy and reliable measurement of giant magneto-thermoelectric effects analogue to the CPP-GMR, showing the possibility of large magnetic control of efficient and macroscopic thermoelectric devices made of lightweight interconnected NW networks integrated into flexible polymer films [15,16,18,20]. As a result, 3D NW networks offer a unique perspective in the emerging field of spin caloritronics, and a real alternative to magnetic nanostructures whose low power output capability limits their applications [21,22]. Moreover, the 3D multilayered NW networks offer a suitable system for the reliable extraction of key spin caloritronic parameters such as the spin-dependent Seebeck coefficient [15,16,20].

Previous works on Co/Cu and CoNi/Cu systems show that the magnitude of the magneto-thermoelectric effect does not differ much from the GMR signal [15,16]. However, there is a strong interest in allowing enhanced control of thermoelectric properties using a magnetic field in such flexible 3D NW network films [15,16,18]. In a recent study on Ni and NiFe nanowires, a strong increase of the magneto-thermoelectric effect compared to the anisotropic magnetoresistance effect is demonstrated for pure Ni [20]. While the Ni/Cu interconnected nanowire networks offer interesting prospects, their fabrication has proved to be very challenging, leading to very low GMR effects [18]. Here, we report on the fabrication and magneto-transport properties of 3D interconnected Ni${}_{x}$Fe${}_{1-x}$/Cu NW networks embedded into flexible polycarbonate (PC) template films with Ni content 0.60≤x≤0.97. The Ni-rich NiFe alloys were chosen because these alloys show promising thermoelectric and magneto-thermoelectric properties, so that a high magnetic modulation of the thermoelectric properties is expected [18]. In addition, the Ni content was not reduced beyond 60% Ni in order to move away from Invar alloys which have poor thermoelectric properties [23].

## 2. Materials and Methods

The 20-µm thick nanoporous PC templates are obtained by a two-step track-etched technique. The film is exposed to a first irradiation step at two fixed angles of −25${}^{\circ }$ and +25${}^{\circ }$ with respect to the normal axis of the template surface. After rotating the PC film in the plane by 90${}^{\circ }$, the second irradiation step took place at the same fixed angular irradiation flux to finally form a 3D crossed cylindrical nanopores network with a mean pores diameter of 80 nm and porosity of about 3%. A Cr (3 nm)/Au (400 nm) cathode is sputtered using an e-beam evaporator on one surface of the template from which the multilayered NWs are grown from single home-made electrolyte solution using a pulsed electrodeposition technique at room temperature (RT) in the potentiostatic mode using a Ag/AgCl reference electrode and a Pt counter electrode following the procedure described in [20]. To adjust the Fe content into the Ni${}_{x}$Fe${}_{1-x}$ layers, the Fe${}^{2+}$ concentration is adjusted in the electrolyte solution. The deposition rates of the Cu and NiFe alloys are extracted from the pore filling time following the procedure described in [24], and the thickness of the bilayers is fixed at about 10 nm with approximately the same thicknesses of FM and Cu layers. The Cu impurity incorporated into the NiFe layers is maintained below 5%, as confirmed by energy-dispersive X-ray analysis (EDX) measurements.

**Figure 1:**
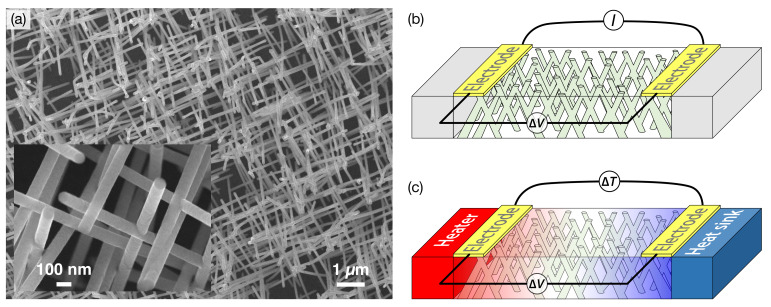
(**a**) Low-magnification SEM image of the interconnected NiFe/Cu NW network film, 80 nm in diameter and about 3% in packing density. The inset shows the branched structure of the NWs, at higher magnification. The diagram shows the experimental set-up for the measurements of (**b**) the electrical resistance and (**c**) the Seebeck coefficient of 3D interconnected NW network film.

At the end of the electroplating step and after complete chemical dissolution of the polymer template, the NW structures form an exact replica of the 3D pristine porous film. Figure 1a shows images of the self-standing interconnected NW structure obtained by field-emission scanning electron microscope (FE-SEM), which demonstrate the NW branching structure and the robustness of the NW architecture. Following the procedure detailed elsewhere [15,18], the resistance *R* and Seebeck coefficient *S* are measured along the macroscopic in-plane direction by locally removing the metallic cathode by plasma etching to create two Au electrodes at the edges of the samples. The electrical contacts are directly made by Ag paint on the metallic electrodes. To measure the electrical resistance, an electrical current is injected between the two Au electrodes while measuring the voltage difference ΔV, as shown in Figure 1b [12,25]. The input power is kept below 0.1 µW to avoid self-heating, and the resistance is measured with a resolution of one part in 10${}^{5}$. To measure the Seebeck coefficient, a temperature difference ΔT, estimated by a differential thermocouple type E, is generated by a resistive heater, giving rise to a thermoelectric voltage ΔV [15,16]. The magnetic variation of the resistance and Seebeck coefficient is measured by sweeping an external magnetic field between ±8 kOe along the in-plane direction of the NW network films. All transport and magneto-transport measurements are performed in the temperature range between 10 K and 320 K.

## 3. Results

**Figure 2:**
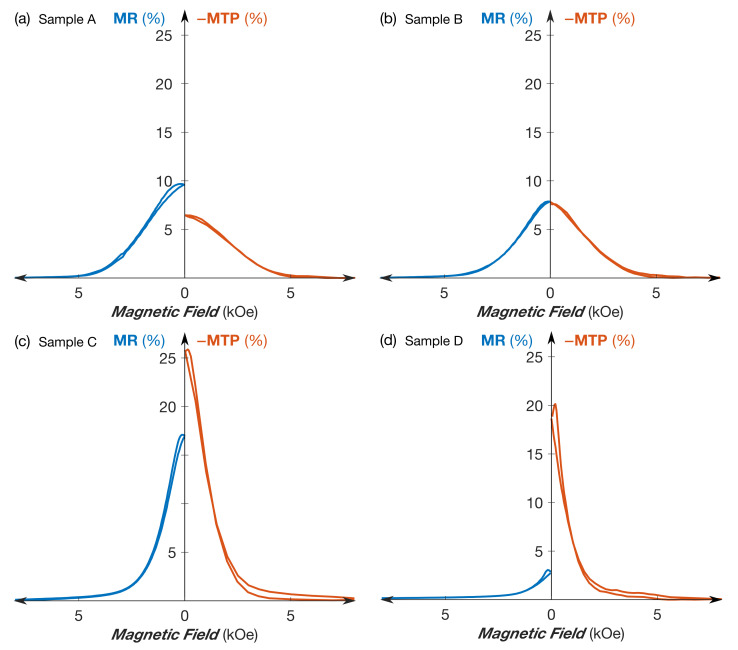
Room-temperature magnetoresistance (left side, in blue) and magneto-Seebeck (right side, in red) curves obtained by sweeping an external magnetic field along the in-plane direction of (**a**) Ni${}_{60}$Fe${}_{40}$/Cu (Sample A), (**b**) Ni${}_{70}$Fe${}_{30}$/Cu (Sample B), (**c**) Ni${}_{80}$Fe${}_{20}$/Cu (Sample C), and (**d**) Ni${}_{97}$Fe${}_{3}$/Cu (Sample D) NW networks.

Figure 2 shows the magnetoresistance and magneto-thermoelectric measurements at RT for various Ni${}_{x}$Fe${}_{1-x}$/Cu NW networks with 0.60≤x≤0.97. Here, MR$\left(H\right)=\left(R\left(H\right)-R_{\mathrm{sat}}\right)/R_{0}$, with R(H) being the resistance at a given external magnetic field value *H*, $R_{\mathrm{sat}}$ the resistance at the saturation field, and $R_{0}$ the resistance at H= 0 and MTP$\left(H\right)=\left(S\left(H\right)-S_{\mathrm{sat}}\right)/S_{0}$, with S(H) the Seebeck coefficient at a given external magnetic field value *H*, $S_{\mathrm{sat}}$ the Seebeck coefficient at saturation field, and $S_{0}$ the Seebeck coefficient at H= 0. Due to the negative values of the Seebeck coefficient in these NiFe/Cu systems, the MTP values are also negative. Therefore, the −MTP curves are shown in Figure 2. The results in Figure 2 show similar field variations for MR and MTP effects for all Ni${}_{x}$Fe${}_{1-x}$/Cu NW networks, despite different amplitudes. This is in contrast to the measurements previously performed on the 3D NiCo/Cu and Co/Cu NW networks, where the amplitudes of the MR and MTP effects are similar at RT [15,16]. As shown in Figure 2a, the MR effect for the Ni${}_{60}$Fe${}_{40}$/Cu is slightly larger than the magnitude of the MTP effect, while very similar magnitudes of the MR and MTP effects are obtained for the Ni${}_{70}$Fe${}_{30}$/Cu sample. When the Ni content in the Ni${}_{x}$Fe${}_{1-x}$ layers is further increased, the MTP effect becomes larger than the MR effect (see Figure 2c,d) for the Ni ${}_{80}$Fe${}_{20}$/Cu and the Ni${}_{97}$Fe${}_{3}$/Cu samples. The largest amplitude of the MR and MTP ratios, which are defined as MR $=\left(R_{0}-R_{\mathrm{sat}}\right)/R_{0}$ and MTP $=\left(S_{0}-S_{\mathrm{sat}}\right)/S_{0}$ respectively, are measured in interconnected permalloy (Py: Ni${}_{80}$Fe${}_{20}$)/Cu NW networks and respectively reached up to 17% and −25%, as shown in Figure 2c. The magnitude of the MTP effect obtained for this multilayered nanowire sample is about five times larger than that previously measured on Py/Cu/Py spin valves, for which a larger MTP effect relative to the MR effect is also reported [26]. For the Ni${}_{97}$Fe${}_{3}$/Cu NW sample, the MTP effect is about 7 times larger than the corresponding MR effect. To our knowledge, such a contrast in the amplitudes of these 2 effects has not been observed before.

**Figure 3:**
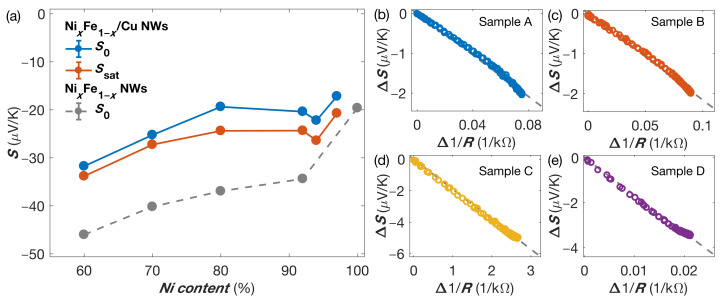
(**a**) Room-temperature Seebeck coefficients at zero magnetic field $S_{0}$ (in blue) and at saturation $S_{\mathrm{sat}}$ (in red) as a function of the Ni content for various Ni${}_{x}$Fe${}_{1-x}$/Cu NW networks. The results previously obtained [20] on homogeneous Ni${}_{x}$Fe${}_{1-x}$ NW networks are also shown for comparison (in grey). (**b**–**e**) Linear variation of $\Delta S\left(H\right)=S\left(H\right)-S_{0}$ vs. $\Delta \left(1/R\left(H\right)\right)=1/R\left(H\right)-1/R_{0}$, illustrating the Gorter–Nordheim characteristics for (**b**) Ni${}_{60}$Fe${}_{40}$/Cu (Sample A), (**c**) Ni${}_{70}$Fe${}_{30}$/Cu (Sample B), (**d**) Ni${}_{80}$Fe${}_{20}$/Cu (Sample C), and (**e**) Ni${}_{97}$Fe${}_{3}$/Cu (Sample D) NW networks. The dashed grey lines in (**b**–**e**) shows the theoretical linear relation given in Equation (1).

The Seebeck coefficients $S_{0}$ and $S_{\mathrm{sat}}$ at RT for various Ni${}_{x}$Fe${}_{1-x}$/Cu NW networks as a function of the Ni content are given in Figure 3a. The Seebeck coefficients previously reported for homogeneous Ni${}_{x}$Fe${}_{1-x}$ NW networks of various composition are also shown for comparison [20]. It is found that, for a given alloy composition, the Seebeck coefficients for the multilayered structures are only slightly smaller than those of the homogeneous NWs. This corresponds well to the behavior expected by Kirchhoff’s law for FM/Cu multilayers in the direction perpendicular to the layers, considering the fact that the Seebeck coefficient of Cu is much lower than the one of the FM [4,20,27]. Figure 3a also shows that the variation of the Seebeck coefficient as a function of the Ni content is very similar for the multilayered NW networks and homogeneous NW systems. As previously reported [18], the room-temperature resistivity for the interconnected Ni${}_{80}$Fe${}_{20}$/Cu NWs was estimated to be about 15 µΩcm in the saturated state, giving rise to a power factor of about 4 mW/K${}^{2}$m. This value is the same order of magnitude as the values obtained for BiTe alloys [28].

Previous works on semiconductor nanowires have shown that it is possible to modulate the thermoelectric properties with a gate voltage [29,30,31,32,33] and achieve power factors of the order of 1 mW/K${}^{2}$m. However, in these systems, an increase in electrical conductivity was accompanied by a decrease in the Seebeck coefficient. For multilayer metallic nanowires, the giant magneto-transport effects lead simultaneously to an increase in the Seebeck coefficient and a decrease in the electrical resistance, which allows a strong increase in the power factor following the application of a magnetic field. Indeed, it has been previously found that a linear variation between the field-dependent Seebeck coefficient S(H) and the inverse of the field-dependent resistance 1/R(H) can be derived from the Mott’s formula for the diffusion thermopower as [34,35,36]:

(1) $$S\left(H\right)=A+\frac{B}{R(H)},$$

where $A=\left(S_{0}R_{0}-S_{\mathrm{sat}}R_{\mathrm{sat}}\right)/\left(R_{0}-R_{\mathrm{sat}}\right)$ and $B=R_{0}R_{\mathrm{sat}}\left(S_{\mathrm{sat}}-S_{0}\right)/\left(R_{0}-R_{\mathrm{sat}}\right)$. This expression is equivalent to the Gorter–Nordheim relation for diffusion thermopower in metals and alloys [37]. Equation (1) can also be re-written as ΔS(H)=BΔ(1/R(H)), where $\Delta S\left(H\right)=S\left(H\right)-S_{0}$ and $\Delta \left(1/R\left(H\right)\right)=1/R\left(H\right)-1/R_{0}$. As shown in Figure 3b–e, such a linear relation between ΔS(H) and Δ(1/R(H)) is observed for all the studied Ni${}_{x}$Fe${}_{1-x}$/Cu NW networks. This highlights the dominant diffusion thermopower for the multilayered Ni${}_{x}$Fe${}_{1-x}$/Cu NW networks. Similar characteristics have already been reported for interconnected Co/Cu [16] and Co${}_{50}$Ni${}_{50}$/Cu [15] NW networks.

**Figure 4:**
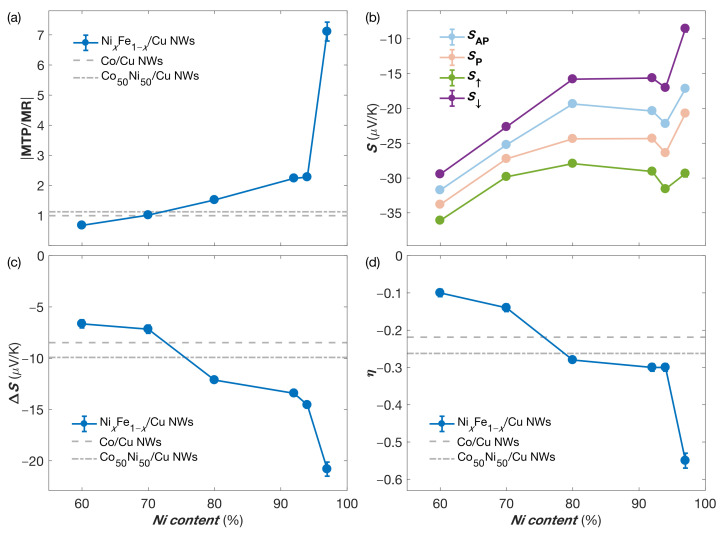
(**a**) |MTP/MR| ratio as a function of the Ni content at room temperature for Ni${}_{x}$Fe${}_{1-x}$/Cu NW networks. The dashed and dot-dashed grey lines correspond to the |MTP/MR| values previously reported for Co/Cu [16] and Co${}_{50}$Ni${}_{50}$/Cu [15] NW networks, respectively. (**b**) Seebeck coefficients in the anti-parallel (AP, in light blue) and parallel (P, in light red) states, together with the calculated Seebeck coefficient for spin up (in green) and spin down (in purple) electrons using Equations (4) and (5) as a function of the Ni content of Ni${}_{x}$Fe${}_{1-x}$/Cu NW networks at room temperature. (**c**) Variation of $\Delta S=(S_{↑}-S_{↓})$ with the Ni content at room temperature. (**d**) Room temperature values of the spin asymmetry coefficient for the Seebeck coefficient η as a function of the Ni content. The dashed and dot-dashed grey lines in (**c**,**d**) corresponds to the ΔS and η values reported for Co/Cu [16] and Co${}_{50}$Ni${}_{50}$/Cu [15] NW networks, respectively.

Figure 4a shows the |MTP/MR| ratio at RT for the Ni${}_{x}$Fe${}_{1-x}$/Cu NW networks as a function of the Ni content. The results in Figure 4a show the strong increase in the MTP effect compared to the MR effect due to the increase in the Ni content in the NiFe alloy layers. Indeed, the |MTP/MR| values range from about 1 for NW networks with Ni content of less than 70% to ∼7 for the Ni${}_{97}$Fe${}_{3}$/Cu sample. For comparison, the reported |MTP/MR| values for interconnected Co/Cu [16] and Co${}_{50}$Ni${}_{50}$/Cu [15] networks are also shown in Figure 4a.

Assuming no spin relaxation, the CPP-GMR effect can be described by the two-current model, in which the resistance of layers and interfaces add and where spin ‘up’ and spin ‘down’ electrons propagate in two independent spin channels with asymmetric resistivities $\rho_{↑}$ and $\rho_{↓}$ [38,39]. Similarly, significantly different Seebeck coefficients for spin-up and spin-down electrons, $S_{↑}$ and $S_{↓}$, are expected because the *d*-band is exchange-split in these ferromagnets, as suggested from previous studies performed on diluted magnetic alloys [40,41]. Assuming that the resistivity and thermopower of the Cu layers are much smaller than those of the Ni${}_{x}$Fe${}_{1-x}$ layers (which is expected for similar thicknesses of NM and FM alloy layers), the resistivity in the parallel (P) configuration of the successive Ni${}_{x}$Fe${}_{1-x}$ layer magnetizations (at saturation field) is given by $\rho_{P}=\left(\rho_{↑}\rho_{↓}\right)/\left(\rho_{↑}+\rho_{↓}\right)$, while the Seebeck coefficient can be expressed as [35]:

(2) $$S_{P}=\frac{S_{↑}\rho_{↓}+S_{↓}\rho_{↑}}{\rho_{↑}+\rho_{↓}}.$$

For an anti-parallel (AP) configuration of the magnetization vector in successive layers, the resistivity is given by $\rho_{\mathrm{AP}}=\left(\rho_{↑}+\rho_{↓}\right)/4$, and the Seebeck coefficient can be written as [35]:

(3) $$S_{\mathrm{AP}}=\frac{S_{↑}\rho_{↑}+S_{↓}\rho_{↓}}{\rho_{↑}+\rho_{↓}}.$$

For multilayered NWs, the magnetization vectors at zero magnetic field are randomly distributed in successive Ni${}_{x}$Fe${}_{1-x}$ layers which, for such thin and numerous FM layers, is expected to be statistically equivalent to the AP configuration [24]. Interestingly, summing and subtracting Equations (2) to (3) lead to $S_{\mathrm{AP}}+S_{P}=S_{↑}+S_{↓}$ and $S_{\mathrm{AP}}-S_{P}=\sqrt{}MR\left(S_{↓}-S_{↑}\right)$, where MR $=\left(\rho_{\mathrm{AP}}-\rho_{P}\right)/\rho_{\mathrm{AP}}$. In consequence, the Seebeck coefficients of spin up and spin down electrons, $S_{↑}$ and $S_{↓}$, can be expressed as [15]:

(4) $$S_{↑}=\frac{1}{2}\left[S_{\mathrm{AP}}\left(1-{\mathrm{MR}}^{-1/2}\right)+S_{P}\left(1+{\mathrm{MR}}^{-1/2}\right)\right]$$

and

(5) $$S_{↓}=\frac{1}{2}\left[S_{\mathrm{AP}}\left(1+{\mathrm{MR}}^{-1/2}\right)+S_{P}\left(1-{\mathrm{MR}}^{-1/2}\right)\right],$$

respectively. Therefore, $S_{↑}$ and $S_{↓}$ can be directly extracted from the experimental measurements of $S_{\mathrm{AP}}$, $S_{P}$, and the MR ratio.

Figure 4b shows the measured values of $S_{\mathrm{AP}}$ and $S_{P}$ at RT for the interconnected Ni${}_{x}$Fe${}_{1-x}$/Cu NW networks. It appears that the general trend is towards a decrease in Seebeck coefficient values following an increase in Ni concentration, as expected for bulk NiFe alloys [23] and in NW networks made of NiFe alloys [20]. Moreover, for all samples, the amplitude of the thermopower can be increased when the magnetization of the NiFe layers is saturated by an external magnetic field. Figure 4b also shows the evolution of the Seebeck coefficients of spin up and spin down electrons $S_{↑}$ and $S_{↓}$ calculated using Equations (4) and (5). An increasing gap between $S_{↑}$ and $S_{↓}$ can be observed due to the increase in Ni content. For the Ni${}_{97}$Fe${}_{3}$/Cu sample, $S_{↑}\approx$−30 µV/K and $S_{↓}\approx$−8.5 µV/K are obtained from Equations (4) and (5). This interesting feature is illustrated by Figure 4c, where $\Delta S=(S_{↑}-S_{↓})$ is presented as a function of the Ni content at RT, revealing a large increase of the amplitude of ΔS when the Ni content is increased towards 100%. The value of ΔS goes from about −7 µV/K for the Ni${}_{60}$Fe${}_{40}$/Cu and Ni${}_{70}$Fe${}_{30}$/Cu samples at RT to about −21 µV/K for the Ni${}_{97}$Fe${}_{3}$/Cu sample. This trend is consistent with the work previously carried out by Cadeville and Roussell [41] on diluted Ni-based alloys, which have deduced very high ΔS values of around −60 µV/K for pure Ni. Moreover, the value obtained for the Ni${}_{97}$Fe${}_{3}$/Cu sample (ΔS∼−21 µV/K) is about two times larger than the spin-dependent Seebeck coefficients measured on interconnected multilayered Co/Cu [16] and Co${}_{50}$Ni${}_{50}$/Cu [15] NW networks, as also shown in Figure 4c.

Figure 4d shows the RT values of the spin asymmetry coefficient of the Seebeck coefficient $\eta =\left(S_{↓}-S_{↑}\right)/\left(S_{↓}+S_{↑}\right)$ for the Ni${}_{x}$Fe${}_{1-x}$/Cu NW networks. The value of η is found to increase with the increasing Ni content in the NiFe alloys. The spin asymmetry coefficient η is found to reach about −0.55 for the Ni${}_{97}$Fe${}_{3}$/Cu NW network, which is the largest value reported until now for multilayered NW networks [15,16,18,20]. For comparison, the values of η reported for Co/Cu [16] and Co${}_{50}$Ni${}_{50}$/Cu [15] NW networks are also shown in Figure 4d. The values for these two multilayered NW networks are about two times smaller than the one of the Ni${}_{97}$Fe${}_{3}$/Cu system. Such a high value for η explains the large increase in the MTP amplitude compared to the MR effect shown in Figure 4a for the Ni${}_{97}$Fe${}_{3}$/Cu NW sample.

The Seebeck coefficients in the AP and P states can also be expressed as:

(6) $$S_{\mathrm{AP}}=\frac{S_{↑}+S_{↓}}{2}\left(1+\beta \eta \right)$$

and

(7) $$S_{P}=\frac{S_{↑}+S_{↓}}{2}\left(1-\beta \eta \right),$$

with $\beta =\sqrt{\mathrm{MR}}=\left(\rho_{↓}-\rho_{↑}\right)/\left(\rho_{↓}+\rho_{↑}\right)$ the spin asymmetry coefficient for the resistivity. Equations (6) and (7) predict that an infinitely large MTP effect might be observed if the product |βη| tends to 1 [15]. Since |β|≤1, this condition can only be fulfilled for systems where $S_{↑}$ and $S_{↓}$ have opposite signs, thus leading to |η|>1.

**Figure 5:**
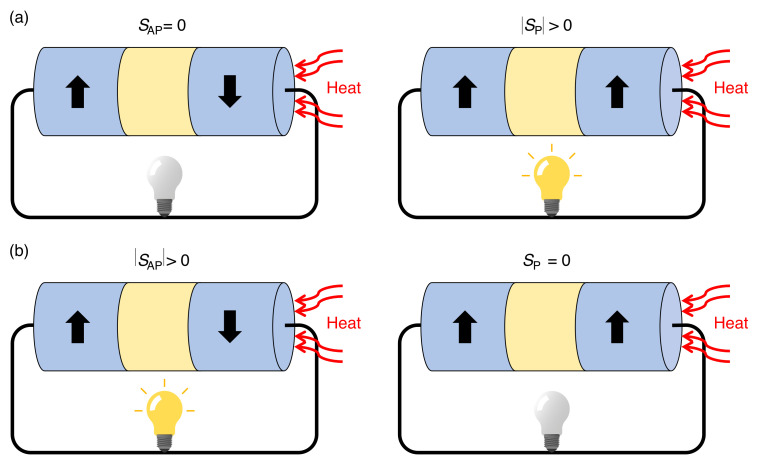
Schematics of a thermoelectric generator based on FM/Cu/FM NWs where (**a**) βη=−1 and (**b**) βη= 1, leading to $S_{\mathrm{AP}}=$ 0 and $S_{P}=$ 0, respectively.

If βη=−1 is satisfied then, at zero magnetic field, no thermoelectric effect occurs ($S_{\mathrm{AP}}$ = 0), whereas thermoelectric power generation is obtained under the application of an external magnetic field. On the other hand, for βη=1 the opposite scenario where $S_{P}$ = 0 is achieved (see Figure 5b). In this case, a thermoelectric power generation is obtained in the absence of a magnetic field, which can be cancelled by an external magnetic field.

On the basis of the results obtained in this work, it appears that NiFe/Cu NW networks with a high Ni concentration are an interesting route for obtaining |η| values larger than 1. The Ni/Cu multilayered system could also be a potential candidate for the observation of a giant MTP effect, since β, although undetermined, is positive and η∼−3 according to Cadeville and Roussel’s estimates for pure Ni [41]. In addition, Farrel and Greig [40] reported $S_{↑}$ and $S_{↓}$ values of an opposite sign in ferromagnetic dilute alloys with the occurrence of a virtual bound state at the Fermi level. Therefore, the fabrication of multilayered NWs with appropriate magnetic layer composition should make it possible to fine-tune the thermoelectric energy conversion with an external magnetic field.

## 4. Conclusions

It was demonstrated that the fabrication of 3D networks consisting of nanostructured nanowires of controlled composition could be carried out simply and reliably by simple electroplating in polymer templates including crossed nano-channel networks. Here, we showed that Ni${}_{x}$Fe${}_{1-x}$/Cu multilayered nanowires (with 0.60≤x≤0.97) could be obtained by a pulsed electrodeposition process from a single electrolyte solution. The nano-composite system obtained by impregnating such a network of interconnected nanowires within a polymer film is flexible and can be easily shaped to macroscopic dimensions. Reliable magneto-electric and magneto-thermoelectric measurements were performed in the direction of the film plane, while limiting electrical and thermal currents along the NW segments of the interconnected NW network. Giant magnetoresistance and magneto-Seebeck effects were measured with amplitudes of up to 17% and −25%, respectively, for the Ni${}_{80}$Fe${}_{20}$/Cu multilayered nanowire network. Although similar field behaviors for magnetoresistance and magneto-Seebeck effects were observed for all samples, the two effects could have very contrasting amplitudes. Indeed, for a Ni content of less than 70 at.%, the MR ratio was found to be slightly higher than or equal to the corresponding MTP ratio. On the other hand, for a higher Ni content, the MTP ratio became higher than the MR ratio and the difference between the 2 amplitudes increased with the increasing Ni content, reaching a factor of 7 for Ni${}_{97}$Fe${}_{3}$/Cu. This effect could be attributed to an increasing difference between Seebeck coefficients for spin up and spin down electrons as the Ni content in NiFe alloys increases. Indeed, the spin-dependent Seebeck coefficient $\Delta S=(S_{↑}-S_{↓})$ increased from −7 µV/K for the Ni${}_{60}$Fe${}_{40}$/Cu and Ni${}_{70}$Fe${}_{30}$/Cu nanowire networks to −21 µV/K for the Ni${}_{97}$Fe${}_{3}$/Cu system. The latter value was the largest spin-dependent Seebeck coefficient directly extracted from measurements on interconnected multilayered NW networks. Similarly, the absolute value of the spin asymmetry coefficient for the Seebeck coefficient |η| increased sharply with increasing Ni content, reaching η = −0.55 for the Ni${}_{97}$Fe${}_{3}$/Cu NW networks. Finally, it should be noted that infinitely large MTP effects were expected for |βη|→1. The results obtained suggest that by optimizing the composition of the magnetic alloy in the electrodeposited FM/Cu multilayered NWs, it is possible to obtain a magnetic switch for thermoelectric generation. Specifically, the Seebeck coefficient could either be increased from zero to a value equal to $S_{↑}+S_{↓}$ or decreased from the same value to zero by applying a magnetic field, thus opening the door to various magnetically controlled temperature management devices or logic devices using thermal energy. It should be noted that the magnetic switch concept for thermoelectricity could be considered on other multilayer nanowire arrays with giant magneto-transport properties.

## Data Availability

The data presented in this study are available on request from the corresponding author.
